# Surface Nanostructuring of a CuAlBe Shape Memory Alloy Produces a 10.3 ± 0.6 GPa Nanohardness Martensite Microstructure

**DOI:** 10.3390/ma13245702

**Published:** 2020-12-14

**Authors:** Carlos Gabriel Figueroa, Víctor Hugo Jacobo, Jacinto Cortés-Pérez, Rafael Schouwenaars

**Affiliations:** 1 Centro Tecnológico Aragón, Facultad de Estudios Superiores Aragón, Universidad Nacional Autónoma de México. Av. Rancho Seco s/n, Col. Impulsora, Cd. Nezahualcóyotl, 57130 Estado de México, Mexico; carlos.figueroa@unam.mx (C.G.F.); jacop@unam.mx (J.C.-P.); 2 Departamento de Materiales y Manufactura, Facultad de Ingeniería, Universidad Nacional Autónoma de México, PIIT, Vía de la Innovación 410, Apodaca, 66629 Nuevo León, Mexico; 3 Departamento de Materiales y Manufactura, Facultad de Ingeniería, Edificio O, Universidad Nacional Autónoma de México. Avenida Universidad 3000, Coyoacán, 04510 Ciudad de México, Mexico; vicjacobo@yahoo.com.mx

**Keywords:** advanced alloy materials, materials synthesis and characterisation, shape memory alloy, surface modification, severe plastic deformation, martensite, nanohardness

## Abstract

Severe plastic deformation (SPD) has led to the discovery of ever stronger materials, either by bulk modification or by surface deformation under sliding contact. These processes increase the strength of an alloy through the transformation of the deformation substructure into submicrometric grains or twins. Here, surface SPD was induced by plastic deformation under frictional contact with a spherical tool in a hot rolled CuAlBe-shape memory alloy. This created a microstructure consisting of a few course martensite variants and ultrafine intersecting bands of secondary martensite and/or austenite, increasing the nanohardness of hot-rolled material from 2.6 to 10.3 GPa. In as-cast material the increase was from 2.4 to 5 GPa. The friction coefficient and surface damage were significantly higher in the hot rolled condition. Metallographic evidence showed that hot rolling was not followed by recrystallisation. This means that a remaining dislocation substructure can lock the martensite and impedes back-transformation to austenite. In the as-cast material, a very fine but softer austenite microstructure was found. The observed difference in properties provides an opportunity to fine-tune the process either for optimal wear resistance or for maximum surface hardness. The modified hot-rolled material possesses the highest hardness obtained to date in nanostructured non-ferrous alloys.

## 1. Introduction

Severe plastic deformation (SPD) is a method for extreme microstructural refinement in bulk materials [1,2,3], producing a nanoscale microstructure which allows to increase the yield strength by a factor up to 3 as compared to conventional processing methods. Recently, SPD has also been studied as a surface modification technique. In analogy with the well-known shot peening treatment, the goal is to increase surface hardness and induce compressive residual stresses to inhibit fatigue crack initiation while maintaining a tough microstructure in the core of the treated part. This is achieved in processes such as Surface mechanical attrition [4,5,6], large strain machining [7], ultrasonic surface modification [8], high-speed pounding [9,10] or sliding contact modification [11,12]. The coaxial configuration proposed by Figueroa et al. [13] for the analysis of adhesive wear induces significant surface modification as well and was, independently, modified as Surface Spinning Strengthening by Ren et al. [14,15,16].

Nanostructuring by plastic deformation under sliding contact conditions was observed long before the research field of SPD-processing was established [17,18]. A formal description and classification of the phenomena involved was given by Rigney in 2000 [19]. Rainforth presented an in-depth analysis of the physical phenomena involved in tribolayer formation and found that strain hardening was the dominating factor, as it determines the achievable subgrain size near the surface [20]. More recently, Rapoport and co-workers have described the relationship between friction, wear, and surface microstructure modification in Cu, Ni Ag, and Al [21,22]. They concluded that friction and wear are determined by strain hardening and dynamic recovery. Higher stacking fault energy (SFE) allows for a more efficient annihilation of dislocations and a lower wear rate. A relationship between SFE and the achievable grain size in SPD was reported earlier by Huang et al. [23]. Figueroa et al. [24] have presented a detailed analysis of substructure and microtexture evolution in copper under sliding contact conditions. Room-temperature recrystallisation occurred, driven by the energy stored in the dislocation substructure created by accumulated small strain events.

The surface modification technique used in the latter work was developed with the dual purpose of studying both SPD and wear [13] and reproduces the phenomena observed in real-world cases of severe adhesive wear in a realistic manner [25]. In earlier studies using this test, it was found that that cold-rolled materials, while harder, show considerably higher wear than recrystallised ones [13,24]. This can be associated to the hypothesis that wear occurs when a critical amount of damage is accumulated in the microstructure [26,27]. For alloys which do not show a shape memory effect, it is understood [24,25,26,27] that small plastic strains are induced by the asperities on the contacting surface. As these asperities affect the surface in a cyclic manner, small strains are accumulated in each step, resulting in total strains which are comparable to the ones achieved in more conventional SPD-processes [1,2,3]. For non-SMAs, the allowable amount of additional plastic strain will be lower in cold-worked materials, because the cyclic plastic deformation is added to the pre-existing one. In these materials, restoration processes such as dynamic annihilation of dislocations and recrystallisation can postpone macroscopic damage by reducing the dislocation content in the material.

Based on the former observations, it was assumed that shape memory alloys (SMAs) may be good candidates for sliding wear applications. Their superelastic (SE) behaviour shows hysteresis which is characterised by the specific damping capacity (SDC) [28]. Theoretically, this hysteresis will allow the transformation of mechanical energy into heat without accumulating plastic strain. In practice, the cyclic loading-unloading of SMAs introduces a small amount of plastic strain which accumulates during the process. This process affects the SDC of the alloy, increases the peak stress during strain-controlled cycling [29,30,31] and influences the fatigue [32] and low cycle fatigue [33] of SMAs. This effect on fatigue confirms the role of dislocation storage as a damage accumulation effect. Although permanent microstructural change may be unavoidable in SMAs under sliding wear, its phenomenology is expected to differ significantly from what is observed in conventional alloys.

Limited studies of sliding wear in SMAs are reported for the Ti-Ni-system [34,35,36], CuZnAl [37], and Cu-Zr [38]. Only the latter paper presents a thorough analysis of the microstructural modification of the alloy during wear, showing that increased wear resistance is associated to martensite–austenite transformation and extensive nanotwinning in the martensite. In the present paper, the surface modification of a CuAlBe-SMA is studied using the coaxial configuration described earlier [13,24]. The focus of the analysis is on microstructure and hardness, but wear data are obtained in the same test and will be discussed in parallel.

## 2. Materials and Methods

The following procedure was used in the experiments:

(a) A Cu-11.4%Al-0.5%Be alloy (wt%) was produced in a high frequency magnetic induction furnace under argon atmosphere and transformed to the β-phase according to the method of Flores-Zuñiga et al. [39]. The martensite start temperature *M_s_* = 253 K was measured on a TA Instruments Q100 differential scanning calorimeter in a temperature range of 233 to 473 K with a ramp of 20 K/min. As a reference, Montecinos et al. [40], analysing a material with the same composition, report *M_s_* = 247 K, *M_f_* = 213 K, *A_s_* = 233 K and *A_f_* = 271 K.
(b) The *β*-transformed billet was sliced to produce samples for different experiments. One set of samples was used in the as cast-state (AC), another was subject to a hot rolling (HR) reduction of 95% at 750 °C, followed by quenching in water at room temperature.
(c) Wear tests and characterisation were performed on as-cast (AC) material and hot-rolled (HR) material. Square test specimens of 2 mm thickness and 20 mm width were cut with a water-cooled diamond-based metallographic cutting disk and polished according to standard metallographic practice (ASTM E3-2017 [41]) to a surface finish with root mean square roughness (*Rms*) equal to 25 nm. Five samples each were tested for the AC and HR states.
(d) Surface modification was performed using a purpose-built coaxial tribometer (details of the configuration and test procedures can be found in [13]). The test consists of pressing an AISI9840 steel cylindrical pin against the surface to be tested. The contact surface of the pin is a spherical cap with radius of curvature of 200 mm. Contact is made by applying a constant normal load on the pin in contact with the test specimen. In the earlier work on Al, AlSn, and Cu, the load was fixed at 100 N. Here, 400 N was used due to the higher hardness of the CuAlBe alloy. Before testing, the pins are cleaned and polished to a *Rms* of 25 nm. Sphericity of the pins is tested periodically and worn pins are discarded. Each test is executed on a new, freshly polished specimen.
(e) The pin is rotated around its own axis at a speed of 60 rpm for 5 min, resulting in 300 cycles per test. A closed-loop feedback system is used to maintain constant load and rotating speed. Load and torque are registered at 0.01 s intervals during the entire test. The test is executed at room temperature. Temperature increase in copper-based alloys is below 2 °C due to the high thermal conductivity of copper and of the aluminium sample holder [24].
(f) Samples were weighted before and after the test with a precision of 0.1 mg. No significant weight changes were found. Therefore, wear damage is quantified by the diameter of the wear track, surface roughness and the torque required to rotate the pin under the applied load.
(g) Topography before and after wear testing was measured with a Nanovea optical profilometer using Chromatic Confocal Technology. *Rms* was calculated after subtracting a 5th-degree fitted polynomial to eliminate the long-range surface topography induced by the test. The *Rms* after the test is a measure of surface damage for samples which do not show significant weight loss and has been shown to correlate with the measured torque [13].
(h) Observations of the worn surface and metallographic sections were performed by polarised light microscopy on a Zeiss Axio Imager A2m reflected light microscope under crossed polarisers. Under these conditions, the cubic DO3 austenite phase, which is optically isotropic, should appear dark, while the monoclinic R19 martensite is optically active and will produce bright features in the micrograph. However, the presence of a naturally formed oxide layer on the alloy causes the austenite phase to appear in blue-green tones, without losing the sharp contrast expected from martensite.
(i) Electron microscopy observations were performed using a Philips XL20 Scanning Electron Microscope (SEM) with an Oxford Instruments Energy Dispersive X-ray Spectroscope (EDS) and the INCA software system.
(j) X-ray diffraction (XRD) was performed on a Rigaku Ultima IV diffractometer using Co Kα radiation in a 2*θ*-range from 10° to 30° in steps of 0.02° with 2.4 s per step.
(k) Atomic force microscopy on polished sections through the centre of the wear zone was performed in contact mode on a Bruker Innova AFM. Nanoindentation measurements were made with a Berkovich indenter with a tip radius of 50 nm on the same AFM. The maximum applied load in nanoindentation was 100 µN with a holding time of 10 s. Post-indentation scans were made in tapping mode to determine the presence of pile-ups/sink-ins. Contact depth was calculated using the Oliver and Pharr method [42,43]. Pile up correction was performed with the semi-ellipse method described by Kese [44,45].

## 3. Results

**Figure 1:**
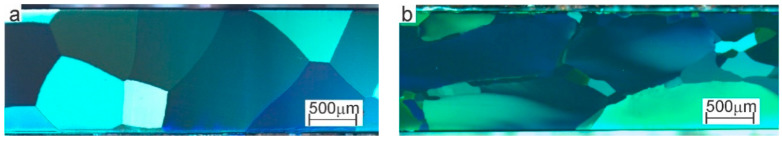
Initial microstructures of the starting materials under crossed polarisers. Grain size is very large. (**a**) As-cast (AC) material. (**b**) Hot rolled (HR) material; brightness variation in the HR-material is due to strain-induced optical anisotropy.

The microstructures of non-modified materials are seen in Figure 1. The entire thickness of the metal sample is shown. Figure 1a shows the AC microstructure, consisting of large equiaxed grains. The HR-sample (Figure 1b), shows elongated grains, some of which show brightness variations caused by the bireflectance induced by internal stresses (see Video S1 in Supplementary Materials). Both effects indicate the absence of recrystallisation after hot rolling.

**Figure 2:**
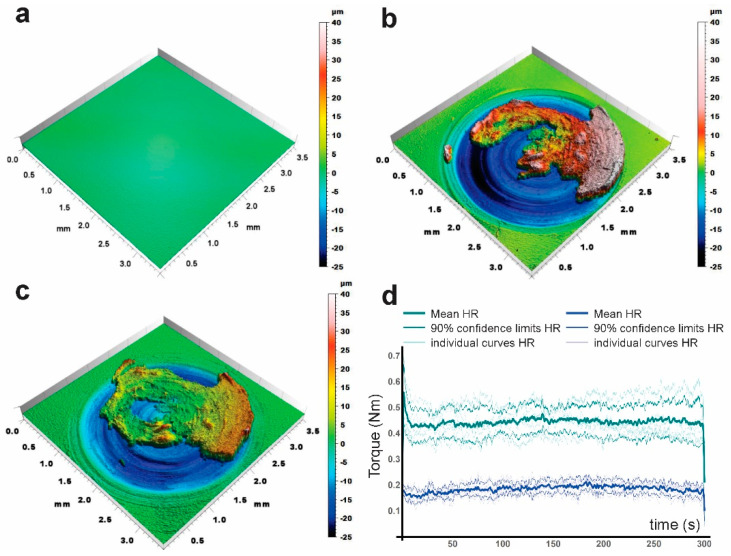
Optical profilometry of the surfaces before and after the test. (**a**): Before testing, (**b**): AC, (**c**): HR) and (**d**) torque measurements during the test, showing the point-by-point averages of 5 tests (5 samples, 30,000 points per curve) and the corresponding point-by point confidence intervals. The 5 individual curves for AC and HR are presented in faint colours as a background.

Representative surface profiles are shown in Figure 2. The measurement on the polished sample is seen in Figure 2a. After testing, the circular wear track can clearly be observed, with a material pile-up toward the perimeter due to the outward plastic flow, produced by the ratchetting phenomena described into detail by Kapoor [27]. Large zones of incorporated wear debris form irregular “mountains” on top of the wear track (Figure 2b for AC, 2c for HR; the images represent the raw measurements without correction for long-range topography). Torque measurements during the experiment are shown in Figure 2d. Torque is much higher in HR-material than in AC. In HR materials, stochastic fluctuations within individual curves (shown as a background) are much higher than in AC materials and differences between individual curves are larger as well. This results in a broader range of variation (as indicated by the confidence intervals) in HR than in AC. Such fluctuations are associated to stochastic micromechanical events such as local adhesion and damage propagation. A smoother behaviour can be associated to better tribological characteristics.

Results of the modification tests are summarised in Table 1. The wear track diameter was measured using SEM on each of the 5 samples for both materials. The torque values for each test were obtained by averaging the 30,000 measurement points obtained for each test, producing 5 time-averaged values for both AC and HR. The mean and standard deviations shown in column 2 are calculated from these time-averaged values. Likewise, a single value of *Rms* is obtained for each of the samples (2^20^ = 1,048,576 measuring points), after correction for the macroscopic topography. The data reported in column 3 represent the statistics of the results for 5 samples. Hardness values were obtained from metallographic sections of two samples from AC and two samples of HR, with 7 indents in martensite and 7 indents in austenite per sample. The values in Table 1 correspond to the non-refined zone, where coarse martensite is formed due to the macroscopic stress field induced by the contacting pin. The average and standard deviation are calculated from the 14 measurements per data set.

The wear track diameter, torque and generated surface roughness are higher in the HR-state. The nanohardness measured outside the wear track shows that austenite is softer in AC than in HR-material, while martensite has the same hardness in both materials. It can be noted that the applied load (100 μN) is relatively low. This value permits a detailed analysis of the gradients close to the surface and allows to measure the hardness of the martensite without influence of the phase boundaries. A disadvantage is that the measured values are subject to the indentation size effect. A recent review of the effect [46], on tempered martensitic steels in the same hardness range as the present alloys, indicate an overestimation of about 25% for a100 μN load as compared to the microhardness. For the AC-Material studied here, the Vickers hardness at a 1 N load was found to be 1.74 ± 0.2 GPa, which is 32% lower than the 100 μN value.

**Figure 3:**
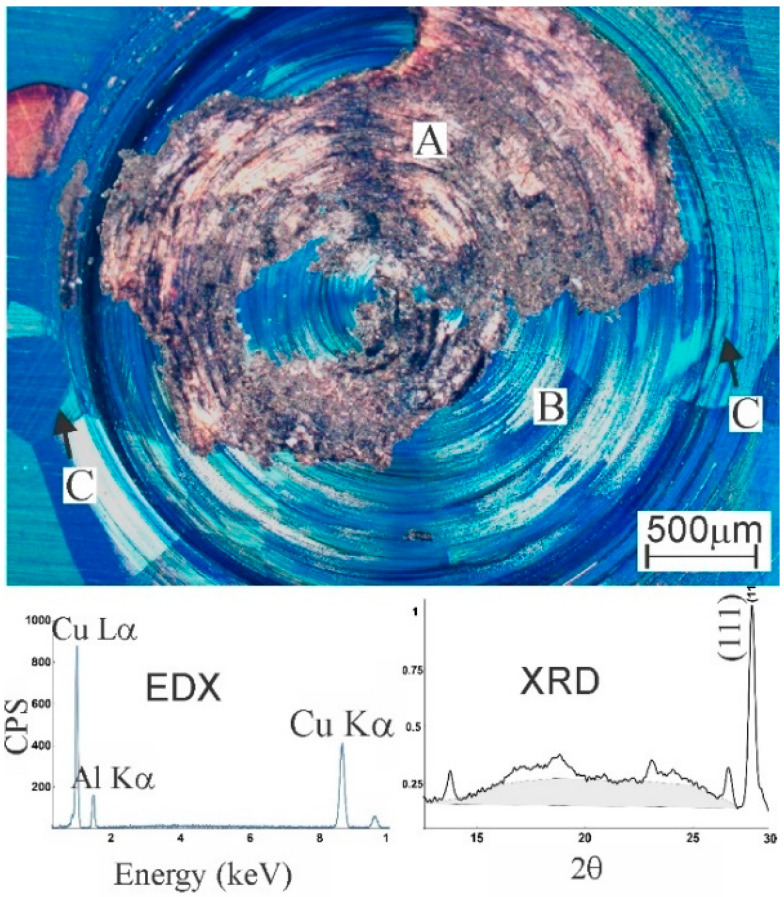
Optical micrograph under crossed polarisers of a HR-sample with an EDX spectrum of zone **A** and XRD of the entire zone. A corresponds to reincorporated wear debris, **B** is the heavily deformed surface, **C** indicates martensite formed around the contact zone.

An optical micrograph of the modified zone in the HR-sample is presented in Figure 3. The zone with higher elevation in Figure 2c is observed in grey (zone A) and consists of reincorporated wear debris. The EDX-measurement shows the absence of Fe, i.e., there is no significant material transfer from pin to substrate. The XRD-spectrum shows a broad hump in the 2*θ*-range from 13° to 27°, indicative of the presence of amorphous material [47], although it cannot be excluded that the effect is due to the surface roughness. Amorphisation was also observed in NiTi-alloys subject to severe deformation [48,49]. Only zone B which will be studied further, as the wear debris is very thin and spread in an irregular manner on top of the severely deformed substrate. Zone B is crystalline but shows effects of shear deformation within the circular track. Outside, the microstructure is modified by martensitic transformation induced by the contact pressure of the pin (arrows C).

**Figure 4:**
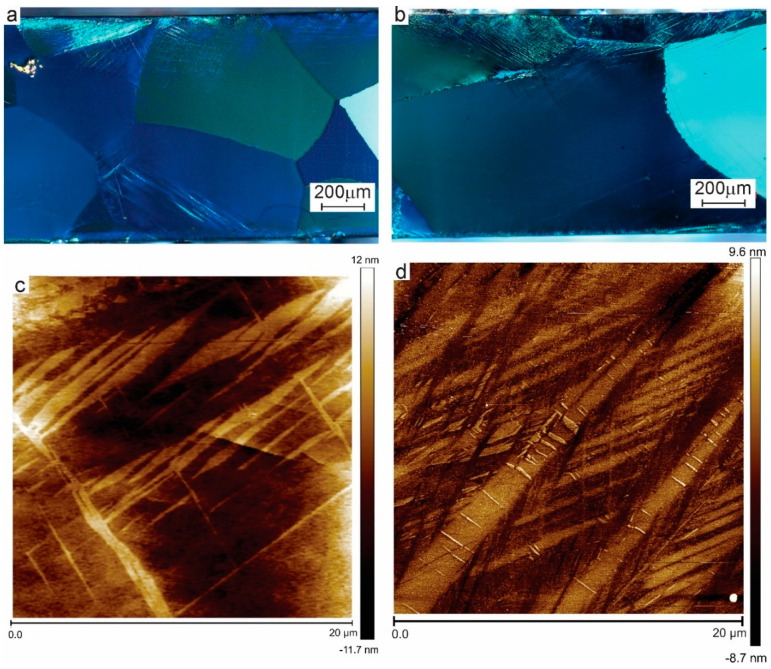
Transverse sections through the modified zones. (**a**) shows the AC material under crossed polarisers, (**b**) shows the same result on HR material, both indicating the presence of very fine intersecting platelets. (**c**) Contact mode AFM observation of nanostructured AC-material and (**d**) contact mode AFM observation of nanostructured HR-material, both representing the upper 20 μm of the modified grains.

Microstructures of the modified zones in AC (Figure 4a) and HR material (Figure 4b) show a myriad of very small elongated features. The optical micrographs are shown to demonstrate the nature and extent of the phenomena involved, but a detailed comparison between AC and HR is better made based on the AFM observations. Figure 4c,d shows AFM-measurements of the ultrafine microstructure of the strongly deformed surface layer in Figure 4a,b respectively. In Figure 4d, a principal martensite variant appears as two diagonal needles. This variant is probably generated by the contact pressure at the start of the test. Between these, at least two sets of very fine, crossed martensite colonies are found with length scales around 100 nm. The AC-microstructure is coarser and shows ragged boundaries. The contrast between microstructural features is lower than in HR.

**Figure 5:**
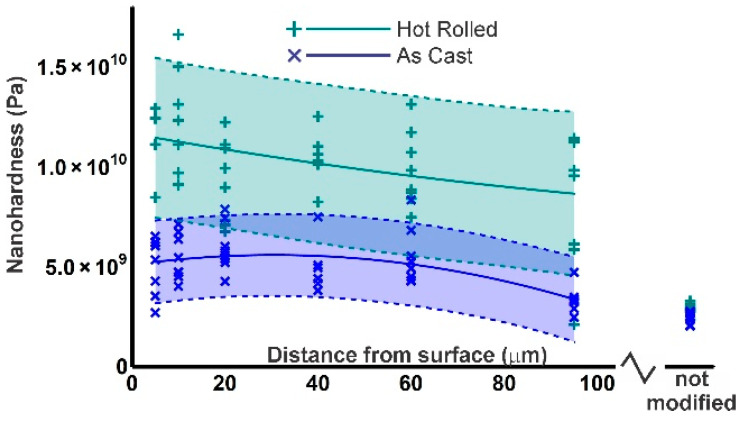
Hardness measurements, with fitting to 2nd degree polynomial and 90% confidence bands.

Figure 5 shows the hardness variation in the modified microstructure. A first important feature is the strong dispersion of the results. This is not an effect of measurement error, as can be seen from the results outside the deformed zone, which have a much lower spread. Even in this zone, the dispersion is not characteristic of experimental error, but is mainly due to the different hardness of differently oriented grains and differences in the shape of the pileups from grain to grain. The dispersion of the data characterises the microscale heterogeneity of the surface layer.

The statistical spread on the data mandates careful statistical analysis. The individual measurements were fitted to a second-degree polynomial by standard least-squares. Ninety percent confidence intervals were calculated based on the results of the corresponding regression analysis and the fitting parameters of the polynomials were evaluated by Fisher-statistics, rejecting parameters with *p*-values > 0.1. The slope in the data for the HR-material was not statistically significant. Hence, all data can be pooled together to obtain an overall average hardness of 10.3 ± 0.6 GPa over the 48 data points in Figure 5. For the AC material, the same conclusion was reached for the slope, but the second-degree term is significant. This indicates a slight decrease in hardness very close to the surface. The average hardness for this material (all data points) is 5.0 ± 0.3 GPa. Notice that the width of the confidence bands in Figure 5 is larger than the confidence intervals on the pooled data, as the latter is based on a higher number of data points.

## 4. Discussion

As a reference to evaluate the results of this work, it can be noted that an increase in hardness from 600 to 1400 MPa was achieved in copper under similar test conditions [24]. In nanotwinned copper, a yield strength of 1000 MPa can be reached [50], corresponding approximately to a hardness of 3000 MPa. Nanotwinned magnesium achieves a yield strength of 600 MPa, as compared to 200 MPa for non-twinned material [51]. A nanostructured copper-niobium composite produced by SPD showed a nanohardness of 4.1 GPa [52] while 3.7 GPa was reported for a copper-vanadium composite [53]. Surface mechanical attrition in annealed pure titanium, TWIP steel and a NiTi SMA produced a maximum hardness of 3.8, 4, and 6 GPa respectively [4,5,6]. In Hadfield steel, shot peening has achieved a maximum hardness of 7.7 GPa [54] and high-speed pounding achieves 8 GPa [9]. In most of these examples, the materials are hardened either by twinning or by martensite formation. The latter mechanism also characterises the CuAlBe-SMA. Only some steels seem to achieve higher hardness than the one presented here. Twelve GPa was measured in the white etching layer of a pearlitic steel subject to rolling-sliding wear [55]. Surface modification of a Fe-1.2%Mn-0.8%Si-0.04%C achieved a nanohardness of 16.2 GPa through the amorphisation of cementite [56]; shock-compressed martensitic steel can reach 19.2 GPa by means of a dispersion of nanoprecipitates [57].

The large spread of the nanohardness measurements is a second important feature of the materials studied here. For the HR-material, the width of the 90% confidence bands is ±4 GPa, for AC, this is ±2 GPa. Microscale heterogeneity is not considered a disadvantage in nanostructured materials formed by SPD [3,58,59,60,61]. In the unmodified material, local hardness variations can be attributed to the elastic [62] and plastic anisotropy of the austenite grains. In the modified material, this effect is enhanced by the fact that the indentation size is comparable to the size of the microstructural features shown in Figure 4d. Different behaviour can be expected if the centre of the indent coincides with the centre of an austenite zone or whether it is close to a martensite or grain boundary [63,64,65]. It shall be noted that the third dimension is invisible to the AFM, so a grain boundary or martensite lath lying below the indented surface cannot be detected. The average value of 10.3 ± 0.6 GPa can be considered as a reasonable first approximation of the hardness of a representative volume element (RVE) of the SPD-modified material, if the RVE is larger than a few mm. 

To explain the high hardness achieved, and the large differences between AC an HR material, two important considerations must be made. Firstly, surface modification SPD processes differ from bulk SPD techniques by the fact that plastic strain is accumulated in a very large amount of small deformation increments under loading-unloading conditions, as first pointed out by Kapoor for railway steels [27] and confirmed by more recent papers on sliding wear [24,25] and surface modification by high-speed pounding [9,10]. Under the experimental conditions used here, the cyclic strain is caused by the individual asperities on the rotating pin which will push a small plastic wedge in front [66,67,68]. In conventional alloys, this accumulation of plastic strain can only be absorbed by plastic deformation and, eventually, recrystallisation [24]. In SMAs, part of the frictional energy is dissipated as heat due to the hysteresis of the hyperelastic behaviour [28], reducing the accumulation of irreversible microstructural modification. 

The latter effect is demonstrated in Figure 4. In Cu [24] or Al-alloys [13], subject to the same process, no trace of the original grain boundaries can be found. The coarse-grained microstructure is transformed into an ultrafine surface zone. Below is a partially refined zone with a morphology which reflects the accumulated shear strain. The latter transforms into a slightly modified zone where the original microstructure is still visible but affected by shear [21,22]. In the present alloys, the original grain boundaries are distorted but clearly visible. Refinement of the surface zone is not achieved by the transformation of a dislocation substructure into new grain boundaries [1,2,3], but by the subdivision of the microstructure into small plates and blocks by cyclic martensitic transformation. This confirms the hypothesis that the damping capacity of SMAs reduces the accumulation of damage under sliding contact conditions.

A second difference between conventional alloys and the present SMA is that the starting material in this work is very hard, even in the as-cast state, due to the solid solution strengthening of Al and Be. These solutes will have a considerable retarding effect on recrystallisation [69]. The HR material is clearly not recrystallised, as evidenced by the elongated shape of the grains, its increased hardness as compared to AC, and evidence of internal stresses in the hot rolled material. Even without TEM-observation, it is evident that absence of recrystallisation means that there is a dislocation substructure within the grains. Dislocations have been shown to lock the martensite, inhibiting the reverse transformation to austenite [70]. The microstructure observed by AFM show a strong geometrical similarity to the ones shown by Tang et al. [71] using TEM-observation on an SPD-processed NiTiCu SMA, although the length scale in the latter observations is much smaller than the one reported here.

To explain the modified microstructure of the AC- material, it can be pointed out that cyclic austenite–martensite transformation under load can lead to austenite refinement and produces jagged boundaries as observed here [72]. Given the large difference in contrast between the martensite boundaries in Figure 4d and the boundaries in 4c, it is reasonable to assume that the entire microstructure is transformed back into austenite and that hardening is due to austenite refinement instead of martensite formation. A single-phased, ultrafine microstructure also helps explaining the lower dispersion of nanohardness data in AC-material as compared to the modified two-phase HR-microstructure shown in Figure 4d.

## 5. Conclusions

As cast and hot rolled CuAlBe-SMA samples were subject to sliding contact surface modification. Hot-rolled material showed extreme hardening which can be associated to the formation of an ultrafine martensite structure, stabilised by the presence of a dislocation substructure which remained in the grains due to the absence of recrystallisation. The nanohardness of this material is higher than what has been achieved by SPD in any non-ferrous alloys and is only surpassed in some nanostructured steels. In the as-cast material, cyclic austenite–martensite transformation can occur. The hysteresis associated to this cycling dissipates part of the mechanical energy induced by sliding contact. This dissipation reduces the energy available to produce wear damage. Hence, AC-material is an interesting candidate for tribological applications, such as electrical contacts subject to sliding wear. On the other hand, if surface strength is the prime concern, as may be the case under fatigue conditions and vibration damping, the HR-material can be developed further.

## Figures and Tables

**Table 1:** Average and standard deviation of measured variables as determined from 5 surface modification tests on AC and 5 tests on HR.

	Contact Track Diameter (mm)	Average Torque (Nm)	*Rms* Roughness (µm)	Austenite Hardness (GPa)	Martensite Hardness (GPa)
CuAlBe AC	2.4 ± 0.1	0.18 ± 0.02	0.64 ± 0.1	2.5 ± 0.3	2.1 ± 0.5
CuAlBe HR	3.2 ± 0.2	0.44 ± 0.06	4.1 ± 0.6	2.9 ± 0.2	2.1 ± 0.3

## Supplementary Materials

The following are available online at https://www.mdpi.com/1996-1944/13/24/5702/s1, Video S1: CuAlBe Optical anisotropy.
